# Opportunities and Challenges Associated with the Pilot Implementation of Clinical Decision Support Systems in a Rural Hospital: A Qualitative Study

**DOI:** 10.1055/a-2581-6236

**Published:** 2025-08-14

**Authors:** Nicki Newton, Adeola Bamgboje-Ayodele, Rowena Forsyth, Lenert Bruce, Steven M. McPhail, Tim Shaw, Sundresan Naicker, Amina Tariq, Melissa T. Baysari

**Affiliations:** 1 Digital Health Human Factors Group, Sydney Nursing School, Faculty of Medicine and Health, The University of Sydney, Sydney, New South Wales, Australia; 2 Department of Biomedical Informatics and Digital Health, School of Medical Sciences, Faculty of Medicine and Health, The University of Sydney, Sydney, Australia; 3 NSW Health, Wagga Wagga, Murrumbidgee Local Health District, New South Wales, Australia; 4 Australian Centre for Health Services Innovation and Centre for Healthcare Transformation, School of Public Health and Social Work, Faculty of Health, Queensland University of Technology, Brisbane, Australia

**Keywords:** clinical decision support systems, pilot projects, hospitals, attitude of health personnel, human-centered design

## Abstract

**Background:**

Despite their potential, Clinical Decision Support (CDS) systems often lack alignment with clinicians' needs and are underutilized in practice. Pilot implementations can help to improve the fit between systems and local needs by engaging users in real-world testing and refinement. Although pilot implementations of CDS have been reported, limited evidence has explored the factors contributing to pilot success.

**Objectives:**

This study aimed to explore the opportunities and challenges associated with the pilot implementation of a CDS system that ultimately did not progress to full-scale implementation.

**Methods:**

We conducted interviews with clinicians, health service managers, and vendors involved in the pilot implementation and use of a mobile application-based CDS, and a dashboard-based CDS in two departments (Emergency and Patient Flow) of a rural Australian hospital. A semistructured interview guide was developed using the Non-adoption, Abandonment, Sustainability, Scale-up, and Spread (NASSS) framework. Interviews were audio-recorded, transcribed, and thematically analyzed.

**Results:**

Analysis revealed four major themes: system performance and design, implementation processes, organizational support and resources, and perceived benefits of the CDS. The pilot implementation allowed for greater user input into the iterative design of CDS in practice, particularly in the Emergency Department (ED), where clinicians had both the capacity and willingness to engage. However, technical issues encountered early in the pilot deterred many users who did not re-engage even after issues were resolved. Although some users remained engaged, they became frustrated as organizational resource constraints meant that critical issues impacting the CDS's clinical utility went unresolved.

**Conclusion:**

Successful CDS pilots depend on the readiness of organizations, departments, and users to engage in pilot activities. Pilot implementations should be pursued in settings where users have both the capacity and willingness to participate in iterative feedback processes and where organizations have sufficient resources to address emerging needs.

## Background and Significance

Clinical Decision Support (CDS) systems provide clinicians with targeted information to assist decision-making at the point of care. 1 Although several studies have demonstrated that CDS can improve patient outcomes, practitioner performance, and care efficiency, many systems fail to achieve these outcomes and are underutilized once implemented in practice. 2 3 4 5 Co-designing CDS with its intended users can result in systems that are useful and easy to use. 6 7 8 9 10 However, not all issues can be predicted or “designed out,” and unanticipated issues will arise once the system is implemented and exposed to real clinical environments. 11 12 13 14

Pilot implementations offer a practical method to determine how a CDS system will fit with existing systems and workflows when used in practice, and engage users in ongoing system improvement. Piloting a system allows for the early detection and resolution of design and workflow issues, provides useful insight into a system's utility, and informs whether and how the system could be implemented at a larger scale. 15 16 17 Though pilot implementations of CDS are common, studies typically focus on CDS performance and outcomes, 18 19 rather than outcomes of the pilot itself (i.e., whether or not to continue to full-scale implementation). Understanding the opportunities and challenges associated with employing pilot implementations of CDS is critical to inform when and how pilots can best be utilized to improve CDS uptake in future. To fill this gap, we conducted a study after the cessation of a pilot implementation of a CDS system in a rural hospital that did not continue to full-scale implementation.

## Objectives

This study aimed to explore the opportunities and challenges associated with pilot implementations of CDS and develop recommendations for future CDS pilots.

## Methods

### Study Design and Reporting

We employed a qualitative case study design, using the Non-adoption, Abandonment, Sustainability, Scale-up and Spread (NASSS) framework to collect and analyze data. 20 The study is reported in-line with the Standards for Reporting Qualitative Research (SRQR). 21

### Clinical Decision Support Intervention and Context

The study was conducted following a proof-of-concept pilot project, funded as part of a broader innovation initiative with the state health departments' digital center of excellence. The initiative aimed to address key challenges in health care delivery through collaborations between health services and industry partners. While limited in-kind implementation support was provided by the vendor and organization, no formal implementation support was scoped or provided in the project.

The pilot leveraged and tested a commercial CDS platform in a rural hospital in New South Wales, Australia. The Emergency Department (ED) was first selected for the pilot due to the willingness of clinical stakeholders within the department to engage. A patient flow-based CDS, while not originally scoped, was quickly deployed to support an urgent emerging need: the COVID-19 pandemic. Doctors were formally engaged in the design of the CDS in the ED, but limited engagement occurred with patient flow nurses. Specific CDS use cases for the pilot were developed iteratively and resulted in different applications being deployed in two departments within the hospital (see Supplementary Appendix for further details [available in the online version only]). These included

1. A mobile application (app) used by doctors in the ED to enhance visibility of patient information, view patient imaging and test results, and support clinical coding.
2. A dashboard with alerts used by nurses in the Patient Flow Department to virtually monitor a small cohort of COVID-19 patients in inpatient and home care settings.

### Setting and Sample

Stakeholders, including clinical users, hospital managers, and vendor staff, involved in the pilot implementation of the CDS system, were eligible to participate. Participants were purposefully sampled to ensure a diverse range of perspectives were captured and to increase information power. 22 23 First, hospital and vendor staff known to the research team were contacted via email and invited to participate. Additional participants were then recruited through snowballing techniques, 24 where participants recommended other staff involved in the pilot implementation of the CDS.

### Data Collection

A semistructured interview guide was developed using the seven domains of the NASSS framework: the condition, technology, value proposition, adopter system, organization, wider system, and embedding and adapting over time (see Supplementary Appendix [available in the online version only]). 20 Questions prompted participants about their experiences with the CDS, the pilot implementation and the removal of the CDS system post-pilot. Interviews were conducted from April to September 2023 by one author (N.N.), a PhD candidate, experienced in qualitative health services research. All interviews were conducted online via video conference, audio-recorded, and transcribed verbatim.

### Data Analysis

All de-identified transcripts underwent independent thematic analysis 25 by two authors (N.N. and A.B-A. or A.T.). Transcripts were uploaded to NVivo 14.23.1 and inductively coded by developing short phrases (codes) that summarized quotes. Codes were deductively mapped to the seven domains of the NASSS framework as they were identified. A coding structure was developed by two authors (N.N. and A.B-A.) following analysis of the first transcript, which was discussed and iteratively updated as analysis progressed. We later grouped codes into themes, according to the core issues that emerged. Interviews continued until thematic saturation was reached, that is, no new overarching themes were identified in transcripts, and sufficient conceptual depth to explain these themes was reached. 26 We compared the perspectives of different participants across the different use cases.

## Results

Twelve participants took part in the study, including six clinicians, three hospital managers and executives, and three vendor staff members ( Table 1 ). Of the six clinicians, four used the ED-based CDS and two used the patient flow-based CDS. Hospital managers and vendor staff were involved in both pilots, however two managers mainly provided insight into the patient flow deployment and one vendor participant mainly provided insight into the ED deployment. Interviews lasted an average of 45 minutes.

**Table 1:** Participant characteristics

Characteristics	Participant ID	*n* (%)
Role		
Nurse	P07, P09	2 (17%)
Doctor	P02, P03, P06, P11	4 (33%)
Hospital manager/executive	P01, P04, P08	3 (25%)
Vendor	P05, P10, P12	3 (25%)
CDS platform involvement		
Emergency department mobile application	P01, P02, P03, P05, P06, P10, P11, P12	8
Patient flow dashboard	P01, P04, P05, P07, P08, P09, P12	7
Gender		
Male	–	9 (75%)
Female		3 (25%)
Clinical experience (y)		
5–10	–	2 (17%)
10–20		1 (8%)
20+		6 (50%)
N/A		3 (25%)

Abbreviation: CDS, clinical decision support.

**Fig. 1:**
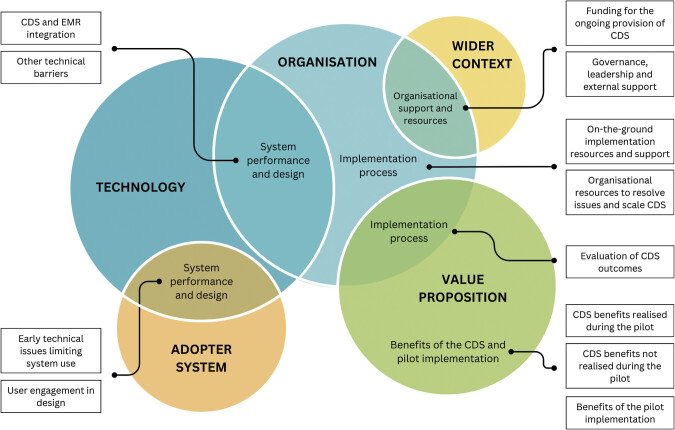
Summary of themes and subthemes aligned to domains of the Non-adoption, Abandonment, Sustainability, Scale-up, and Spread (NASSS) framework. CDS, clinical decision support; EMR, electronic medical record.

The scope of the ED-based CDS was described to evolve throughout the course of the pilot, while the patient flow dashboard was not scoped in the original pilot but was quickly deployed to support the urgent needs of the pandemic. ED doctors described using the app for 3 to 6 months, whereas patient flow nurses described using the dashboard for only 2 weeks. We identified four major themes, including system performance and design, implementation processes, organizational support and resources, and benefits of the CDS. Themes, subthemes, and related NASSS domains are described below and presented in Fig. 1 . Barriers and facilitators to individuals' adoption of CDS within each department and ongoing adoption of CDS at the organizational level are described within themes below and presented in alignment with NASSS domains in Supplementary Table S1 (available in the online version only).

### System Performance and Design

#### Early Technical and Design Issues Limiting Use (Technology and Adopter System Domains)

Clinicians described several technical issues that hampered use of the CDS shortly after it was made available to users. A vendor participant reported that the ED-based app faced issues with data storage and servers crashing, which affected system performance. Though many of these issues were believed to be resolved throughout the pilot, participants reported that several users had already stopped using the system by that stage. An ED doctor reported that “initially, we would have had 10 or 15 users… probably 50% of them dropped off fairly rapidly. I think there were a number of people that were happy to give it a try but… perhaps couldn't see beyond its current capability” (P02).

Similar technical challenges were reported with the patient flow dashboard, particularly regarding its integration with remote monitoring devices. For example, oxygen saturations recorded in the dashboard were reported to be inaccurate, which resulted in nurses having to constantly escalate by calling patients to confirm readings. Alerts were perceived to fire in the dashboard inappropriately, such as when a patient removed the device or when it was out of Bluetooth range, resulting in alert fatigue. Participants felt that these technical issues were not addressed quickly enough after the system went live, contributing to nurses' lack of trust in the system. This trust was perceived as difficult to rebuild: “…you can't make any mistakes with the implementation. Because if you do then the trust is broken and the clinicians just won't use it” (P04).

#### User Engagement in Design (Technology and Adopter System Domains)

ED doctors and vendor staff described working together throughout the pilot to enhance the app's design and clinical utility. Though some ED doctors stopped using the app early on, others continued to use the system as they wanted to “improve the product and deliver what we really wanted it to… improving our processes and our patient care” (P02). All ED doctors reported that the app was easy to use and that they could easily provide feedback to the vendor, who were quick to respond and resolve most design issues.

Conversely, patient flow nurses perceived they had insufficient input into the dashboard's design and development, contributing to their lack of trust in the system. Although participants perceived a clinical need for tools to improve remote monitoring of patients during the COVID-19 pandemic, they found the dashboard to be “overly complex and probably overly engineered for the COVID patient cohort, which don't need 24/7 monitoring” (P04). While participants felt that the system could have been adapted to meet their needs, the instability, unfamiliarity, and urgency of the pandemic created pressure to implement a solution as quickly and efficiently as possible. Clinicians in this setting were also perceived to be less willing to participate in the design process or use the system because “there was no guarantees beyond the three months or six months. So a lot of the time people felt like they were putting in work for just a real short term thing” (P04).

#### Clinical Decision Support and Electronic Medical Record Integration (Technology and Organization Domains)

Ongoing technical issues relating to the integration of the ED-based app and hospital-wide electronic medical record (EMR) were perceived to hamper clinicians' use of the system over time. This included issues with the data feed from the EMR to the app that resulted in out-of-date data, as well as a lack of “write back” integration from the app to the EMR. Doctors consequently needed to refer to the EMR as the source of truth after checking the app, essentially duplicating work. For example, when viewing critical test results on the app, doctors described that “by not having write access, the parent system had no way of knowing whether you'd looked at the information or not” (P02). Resolving this issue was perceived to be crucial for realizing expected benefits and addressing barriers to use. Although write-back integration was technically possible, it was not scoped within the pilot and was unable to be addressed due to organizational approval and resourcing constraints.

#### Other Technical Issues (Technology and Organization Domains)

Other technical barriers to using the app in the ED included the need for a separate mobile device (i.e., not a personal device) to use the app that was not used for any other purpose, as well as permissions and sign-on issues that, over time, led clinicians to discontinue use of the system. For example, doctors described forgetting to charge the device, or not bothering to reinstate logins after experiencing sign-on issues. Participants described “black holes” in the Wi-Fi network throughout the ED and periods where the 4G network would “drop out,” causing a lag in updating of patient information, which limited system use.

### Implementation Process

#### On-the-Ground Implementation Resources and Support (Organization Domain)

In the patient flow department, participants perceived there to be limited implementation support and resources, where a lack of change management, onboarding, and training hindered the dashboards' use. A patient flow nurse explained: “If we had someone to say, ‘Okay, this is the device, this is how it's used. This is the database, this is how you interpret it. If you’ve got any questions, this is how we escalate'.… there was no real model of care around it.” (P09). This led to concerns around patient safety, where care delivery was described to “feel unsafe” (P09). Participants described the lack of implementation support to be especially problematic, given that they were not involved in the dashboard's design and therefore were unfamiliar with it. Vendor participants attributed this issue to the pilot's shift from a planned preproduction demonstration to a live production trial with real patients, where the necessary resources were not available to support the expanded scope.

In contrast, despite describing there to be little formal training or implementation support, ED doctors felt comfortable using the system. Those who were involved in the CDS design described championing the system, promoting it among other clinicians, and teaching them how to operate it.

#### Organizational Resources to Resolve Issues and Scale Clinical Decision Support (Organization Domain)

Several issues were described to stem from a lack of financial support, planning, and staffing for the pilot. This included a lack of project management personnel that participants perceived would have been assigned if the CDS were implemented following a traditional implementation approach. One participant from the vendor organization described there to be “no one else there except the doctors, and some time from one of the IT (Information Technology) staff to make some of these difficult sort of technology changes that we were working through” (P10). This caused issues, for example, where no IT specialists at the organization had the appropriate skillset to complete the write-back integration from the app to EMR. Similarly, participants described the spread of CDS to other users, such as specialists in inpatient departments, being blocked due to a lack of organizational funding and resources to expand the scope of the pilot.

#### Evaluation of Clinical Decision Support Outcomes (Organization and Value Proposition Domains)

Participants perceived there to be a lack of formal evaluation and limited time to establish and demonstrate the benefits of the pilot. Consequently, the value of the system was unable to be demonstrated to leadership in the organization to justify ongoing costs, or to clinicians on the ground to drive their ongoing use of the system. One ED doctor described: “if they had good outcomes from it, we hadn't really had feedback as end users… we never really knew the outcome… we don't know what we achieved” (P11).

### Organizational Support and Resources

#### Funding for the Ongoing Provision of Clinical Decision Support (Organization and Wider Context Domains)

A lack of funding for the ongoing provision of the CDS was raised by several participants. This was often perceived as due to the organization being a rural health service. Some participants perceived that the organization did not have the budget available to support ongoing investment in the CDS, which they felt ultimately led to the decision not to continue with the system post-pilot. Organizational leadership described it to come down to a “cost versus benefit decision. The time when the decision had to be made, we could not make a compelling argument to continue on” (P01). Given that the COVID-19 pandemic arose during the pilot, some participants also attributed the resources required for the pandemic to be prioritized over resources required to support the ongoing provision of the CDS.

#### Governance, Leadership, and External Support (Organization and Wider Context Domains)

Most ED doctors perceived there to be good support for their use of the mobile app from departmental and executive leadership. However, participants suggested the project lacked appropriate governance structures within the hospital to support required approvals, for example, to resolve integration issues. One staff member was frequently mentioned by participants as the driver of the pilot at the organizational level. However, this individual resigned during the pilot, and participants perceived this loss to be a major barrier to the project's success: “you lose one person and you don't have anyone who's driving that, who knows all the details through the organization and how to pull it together” (P05).

A lack of continued support from the state government agency responsible for the innovation initiative was also raised by vendor participants as a challenge for the ongoing provision of the system at the hospital. This was perceived to be particularly important given resources available at the local level were limited.

### Benefits of the Clinical Decision Support and Pilot Implementation

Participants described a range of benefits resulting from CDS use, as well as potential or future benefits that were expected but not realized during the pilot. Additionally, benefits resulting from the pilot implementation process were described.

#### Clinical Decision Support Benefits Realized during the Pilot (Value Proposition Domain)

ED doctors perceived the app to improve communication and collaboration between staff, increase mobility within the department, increase accessibility to the EMR, and improve efficiency during handover. In the patient flow department, participants described instances where the dashboard had identified deteriorating patients, which enabled nurses to quickly respond. Vendor and organizational leadership participants highlighted the real-time extraction of data from the EMR for CDS use as a valuable capability demonstrated during the pilot, noting that this had not been achieved previously. Unanticipated benefits were also raised, such as retaining access to patient information during a period of unexpected EMR downtime.

#### Clinical Decision Support Benefits not Realized during the Pilot (Value Proposition Domain)

Potential benefits of the CDS systems, such as improved workflow efficiency, patient experience and safety, and cost-effectiveness, were recognized but perceived as not being realized in the pilot due to technological, implementation, and organizational barriers. Participants who described using the system for a longer period mostly stated that their motivation to continue using it was due to these larger-scale benefits that they expected the system would deliver. However, they described using it less over time as these expectations were not met.

Participants who held leadership roles perceived the CDS' value to remain unrealized as it was not scaled across users, departments, and hospital sites, describing that “the benefit lies in scalability. Having it in just one department in the hospital was never going to deliver the full benefits of the system” (P01). Limited scaling of the system was attributed to a lack of marketing across different users, departments, and hospitals within the health district.

#### Benefits of the Pilot Implementation (Value Proposition Domain)

Participants described benefits arising from the pilot process, such as facilitating the broader development of clinicians' skillsets and creating networks for the health service and individual clinicians with healthcare innovators. Those from the vendor organization described securing new contracts following the pilot, where “the genesis of what's in there (the contracted product) came from some of that work” (P12).

## Discussion

This study provides critical insights into the opportunities and challenges associated with pilot implementations of CDS systems. Though some opportunities were identified, the current study mostly revealed challenges. We found limited added value for ED clinicians (due to unresolved technical issues), and for organizational leadership (due to the limited use and scale of the CDS across the organization, and the departure of the pilots' champion), as key challenges contributing to the removal of the CDS system post-pilot. Technical issues, compounded by a lack of user input into CDS design and instability during the COVID-19 pandemic, led nurses to reject the patient-flow CDS shortly after its implementation. These issues were created or amplified by the limited organizational, implementation, and evaluation resources dedicated to the pilot. Though previous studies have demonstrated that CDSs' level of integration, 27 perceived value, 28 29 30 and user input 31 can impact clinicians' acceptance of a system, this study underscores their criticality by providing evidence that CDS implementations may not be sustained when such needs go unmet.

Although the pilot did not continue to full-scale implementation, our research suggests that pilot implementations can provide valuable opportunities to involve clinicians in ongoing CDS design and to make a system's value visible within and beyond an organization. For example, the pilot facilitated lasting networks between health services and innovators and demonstrated the technical feasibility of extracting EMR data for use in CDS systems. These outcomes laid the groundwork for further initiatives that were pursued beyond the pilot. While some benefits of both CDS were observed during the pilot, clinicians perceived these to be of lower impact than those that were not realized, as they either had minimal direct effect on their daily work or were overshadowed by significant barriers to system use. Like previous research, we emphasize that CDS systems should be designed to deliver clear, direct benefits to end-users while also addressing broader organizational and patient outcomes, with efforts made to effectively communicate these broader benefits to clinicians. 32

Users who were heavily involved in CDS design, which is, in the ED, found the system to be intuitive and recognized its potential to deliver valuable clinical benefits. However, key issues raised by users went unaddressed in the pilot due to organizational approval bottlenecks and resource constraints, preventing these benefits from being realized. Future pilots should therefore implement robust governance structures to facilitate the timely resolution of user-identified issues and enhance responsiveness to evolving needs. 33 34 35 Furthermore, while many ED clinicians were willing and able to be involved in the pilot, patient flow clinicians lacked the capacity to fully engage due to the urgent demands of the pandemic. This highlights the importance of matching CDS systems and implementation approaches to the needs, capabilities, and capacity of organizations, departments, and users. CDS implementers may additionally consider evaluating the readiness of organizations and users to engage with the technology and pilot process. 36 37 38

Another key finding was that negative user experiences early after implementation played a crucial role in shaping the long-term success of the CDS system, providing insight into why previous research has found system usage can remain low even after initial issues are resolved. 12 39 Our research suggests that, even in pilot implementations, CDS systems should be deployed with a sufficient level of maturity to prevent lasting user disengagement. Future pilots may wish to purposefully select a small group of early adopters who can participate in the iterative design process and pave the way for broader adoption by later users, who may prefer a more stable system that delivers immediate benefits. 40

Lastly, our study expands on the unique challenges faced by pilot implementations in health service environments. 15 16 17 41 Like previous research, we found that clinicians viewed the pilot as a temporary initiative, which disincentivized their engagement with the system and design process. 15 Additionally, the conceptualization of the project as a pilot meant that limited resources and organizational structures were established to support it. The pilot was therefore found to be particularly vulnerable to organizational changes, such as the departure of key personnel and shifting priorities during the COVID-19 pandemic. We recommend pilots be supported by robust project and change management, evaluation, and organizational oversight to mitigate these risks and secure the strong and sustained commitment needed for CDS success. 42 43 44

Limitations of this study included the relatively small sample size, particularly in certain categories of participants, such as users of the patient-flow dashboard, and the likelihood that those who chose to participate in the research were individuals more engaged with or invested in the technology. As a result, some nuances in user experiences may not have been fully captured. Additionally, our focus on a single rural hospital may limit the generalizability of findings to dissimilar settings. For example, urban hospitals are likely to have greater access to IT specialists and resources, which could mitigate technical challenges such as those relating to system integration and connectivity infrastructure that were faced in the current study. Despite this, our findings are strengthened by the information power of our sample, 23 alignment with previous research conducted on CDS and pilot implementations in other contexts, and alignment with the NASSS framework. Another limitation is that we relied on retrospective accounts from participants, which may have introduced recall bias. 45 However, this provided an opportunity to explore what unfolded following pilot cessation, offering valuable insights into its longer-term impacts. Future research may wish to validate and expand on our findings by prospectively studying CDS pilots in differing contexts and incorporating quantitative usage metrics to evaluate actual use of CDS during pilot implementations.

## Conclusion

While pilot implementations can offer a useful approach to engage users in CDS design in practice and expose the potential benefits a CDS can offer, pilots also present unique challenges and should be approached with caution. To be effective, pilot implementations must be supported by adequate organizational and implementation resources to ensure that iterative design based on user feedback can be fully addressed. CDS implementers should carefully weigh the potential benefits and challenges of utilizing a pilot approach and consider the readiness and fit of the organization, department, and its users for the technology and implementation approach.

## Clinical Relevance Statement

This study provides practical insights for health care organizations and vendors looking to implement CDS systems. CDS implementers can leverage insights from the opportunities and challenges identified to select the most suitable implementation approach. By applying recommendations, they can optimize future pilot implementations and increase the likelihood of achieving successful and sustainable adoption of CDS systems.

## Multiple-Choice Questions

1. What role did iterative design play in the pilot implementation of CDS in the study?
  - It ensured that no changes were needed post-pilot.
  - It limited the ability of clinicians to influence system design.
  - It allowed for the system to be refined based on real-world user feedback.
  - It guaranteed immediate adoption across all hospital departments.
  **Correct Answer** : The correct answer is option **c** . Iterative design was highlighted in the study as a key opportunity for pilot implementations. It allowed clinicians to provide feedback on the CDS system, which informed refinements to better align with their needs.
2. What happened during the early stages of implementation that impacted long-term success of the CDS in the study?
  - Clinical champions actively promoted the CDS among other potential users.
  - Many users disengaged from the CDS after encountering technical issues.
  - All technical issues were successfully resolved.
  - Early adoption ensured sustained engagement with the CDS by clinicians.
  **Correct Answer** : The correct answer is option b. The study found that early technical issues significantly impacted many clinicians' perceptions of the system, leading to disengagement that persisted even after issues were resolved.
3. Which of the following were not recommended by the study for future pilot implementations of CDS?
  - Implement appropriate governance structures
  - Find an organizational champion who can drive the pilot
  - Support the pilot with project and change management resources
  - Evaluate the readiness of users and organizations to engage
  **Correct Answer** : The correct answer is option b. While organizational champions can be valuable to support pilot projects, our research highlighted the risks of overdependence on individuals.
