# Supplementary material for: Opportunities and Challenges Associated with the Pilot Implementation of Clinical Decision Support Systems in a Rural Hospital: A Qualitative Study
Source: Appl Clin Inform. 2025 Aug 14;16(4):777–85. doi: 10.1055/a-2581-6236 (PMC12352988; doi:10.1055/a-2581-6236)
Supplement: Supplementary file 1 — Supplementary Material [file 10-1055-a-2581-6236_26798738.pdf]

Supplementary Appendix

Intervention and Pilot Description

The project was funded as a proof-of-concept pilot in August 2018, aiming to mobilize data to improve the safety and quality of care during a patient's stay in the hospital. The pilot originally intended to demonstrate Clinical Decision Support (CDS) functionality within a test environment only. However, in initial meetings with project leadership, it was agreed that real-world testing with clinicians in a clinical environment was needed to prove the functionality. The CDS systems deployed utilized the same platform, developed by a single vendor.

Emergency Department Clinical Decision Support

Vendor staff conducted site visits to the hospital to collect informal feedback from clinicians before and during the pilot. Additionally, they held two formal workshops with clinicians to identify the minimum dataset required and gather feedback on a live prototype before the pilot, and another two formal design workshops with clinicians during the pilot. Emergency Department (ED) clinicians had direct access to the vendor via phone call to request minor changes or report issues during the pilot.

A mobile application (app) was deployed and piloted with ED doctors, which aimed to provide enhanced access to patient information, track patient activity, and notify doctors of critical test results within the ED in real-time to reduce cognitive load and increase mobility, and to enhance the accuracy of clinical coding.

- **Integration**: The app provided mobile access to the electronic medical record (EMR) (Cerner), receiving real-time data through one-way integration (from the EMR to the app).
- **Key features**
  - Ability to see all key elements of the EMR on a mobile device
  - Ability to track patients in the ED and quickly access patient lists
  - Ability to have quick access to radiology results on a mobile device
  - Ability to quickly review past visits for a patient on the mobile device
  - Ability to identify key conditions from past hospital encounters through natural language processing
- **Timeline**: The pilot implementation of the app occurred between June 2019 and February 2022.

Patient Flow Department Clinical Decision Support

A dashboard system with embedded alerts was deployed and piloted with patient flow nurses, which aimed to support the monitoring and management of patients with COVID-19 and detection of potential deterioration. Online meetings were held with operational and clinical staff prior to deployment.

- **Integration**: The dashboard received real-time data from the EMR (Cerner) and remote monitoring devices that provided continuous (24/7) monitoring of a small cohort of patients who were diagnosed with COVID-19 in inpatient and home settings.
- **Key features**
  - Ability to view patient details using data (e.g., name, age) from the EMR
  - Ability to continuously monitor a series of vital signs (e.g., blood pressure, respiratory rate, skin temperature, heart rate, and oxygen saturation) from remote monitoring devices
  - Ability to receive alerts from the dashboard based on incoming data from remote monitoring devices
- **Timeline**: The pilot implementation of the dashboard occurred between April 2020 and September 2021.

 © 2025. The Author(s).

Interview Guide

General Questions about the Clinical Decision Support

1. What decision support tools/products did you use? E.g., app, dashboards, logic
   a. What device/s did you use it on?

Condition

2. What illness/es or condition/s was the decision support tool/s designed for?

Technology/Technologies

3. How did the tool/s support your work? i.e., what tasks did you use it for?
   a. Were there some tasks that the tool supported better than others?
4. What did you think of the decision support system's performance and dependability?
   a. How well do you think it supported the tasks for which it was designed?
   b. What worked about the tool/s? What did you like about it?
   c. What didn't work about the tool/s? What didn't you like about it?
5. How did the decision support tool/s integrate with other systems?
   a. What other systems are used in your department?
      i. For example, EMR, other devices (remote monitoring, etc.), pathology

Adopter System

6. Who were the main users of the decision support?
7. How were you and other users involved in the design of the decision support?
8. How usable and acceptable did you think the decision support tool/s were to you and other users? E.g., trust, ease of use, time taken, workflow fit
   a. How often did you use the tool? Why or why not?
      i. Were there any differences in how you used the tools over time?
9. How do you think the decision support tool changed the way health care was delivered?

Value Proposition

10. Can you give me an example of how the decision support tool added value to patients?
    a. What was valuable about it?
11. Can you give me an example of how the decision support tool added value to you and other clinicians?
12. Can you give me an example of how the decision support tool added value to your organization and the healthcare system?
13. Did the technology generate any negative value (i.e., any costs, risks, or disruption) for you or other stakeholders? Did the benefits of the tool outweigh any risks?

Organization

14. What do you think of the organization's overall capacity to take on technological innovations? E.g., leadership, slack resources, previous experience, risk-taking
    a. What about at the local departmental level?
15. How did your organization go about implementing the decision support tool? Prompts:
    a. How ready do you think your organization was for the decision support tool when it was implemented?
    b. How were you trained to use the tool?
    c. What supporting resources (e.g., guidelines, procedures, education) were you provided with?
    d. Were leadership or other clinicians supportive of its use?
16. Did any organizational routines, pathways, and processes change to accommodate the decision support tool? How?

 © 2025. The Author(s).

The Wider Context

17. What were some of the external challenges at the time of the implementation? E.g., political climate, professional, clinician, legal, patient, organization

Embedding and Adapting over Time

18. How long did you use the decision support tool for?
19. Could you or other users provide feedback on the decision support tool following its implementation? How?
20. Were there any changes made to the decision support tool following its implementation?
    a. Did these changes accommodate yours or other users' needs? How?

Pilot Cessation

21. What do you think are the main reasons that caused the decision support tool to be removed?
    a. Were there any external changes that contributed to it?
    b. Organizational changes?
    c. What caused you to stop using the decision support tools?
22. Is there anything you would do differently, or encourage others to do, if a new decision support system were being implemented? i.e., lessons learnt
23. Do you have any other comments about the decision support tool or pilot?

 © 2025. The Author(s).

Supplementary Table S1 Barriers and facilitators mapped to Non-adoption, Abandonment, Sustainability, Scale-up, and Spread domains

Domain	Theme	Emergency department	Patient flow	Organizational adoption
Technology	Integration and interoperability	–	–	–
	Alert fatigue	N/A	–	N/A
	System maturity	–	–	N/A
	User engagement in design	+	–	N/A
	Changes to made technology	±	–	N/A
	Fit with clinical need	+	–	N/A
	System performance, usability, and satisfaction	±	–	N/A
Organization	Implementation support	+	–	N/A
	Departmental stability	N/A	–	N/A
	Evaluation and feedback	–	N/A	–
	Trial nature of implementation	N/A	–	–
	Organization-wide technology infrastructure	–	N/A	N/A
	Fit with workflows	–	–	N/A
	Departure of pilot champion	–	N/A	–
	Organizational resources, structures, and leadership support	±	N/A	–
Value Proposition	Benefits of the system	+	+	+
	Potential benefits of the system	+	+	+
	Did not deliver expected value	–	–	–
	Benefits of the pilot implementation	+	N/A	+
Adopter System	Champions and super users	+	N/A	N/A
	Familiarity	N/A	–	N/A
	Trust	+	–	N/A
Wider Context	Impact of COVID	N/A	–	–
	Government support	N/A	N/A	–

Note: Barriers (–) and facilitators (+) to individuals' adoption of Clinical Decision Support (CDS) within each department and ongoing adoption of CDS at the organizational level.

 © 2025. The Author(s).
